# Sex Differences in *Drosophila* Somatic Gene Expression: Variation and Regulation by *doublesex*

**DOI:** 10.1534/g3.116.027961

**Published:** 2016-04-19

**Authors:** Michelle N. Arbeitman, Felicia N. New, Justin M. Fear, Tiffany S. Howard, Justin E. Dalton, Rita M. Graze

**Affiliations:** * Biomedical Sciences Department and Center for Genomics and Personalized Medicine, Florida State University, College of Medicine, Tallahassee, Florida 32306; † Department of Molecular Genetics and Microbiology, University of Florida, Gainesville, Florida 32610-0266; ‡ Department of Biological Sciences, Auburn University, Alabama 36849

**Keywords:** sex determination, Drosophila, sex hierarchy, doublesex, transcriptome, gene expression, sex bias, RNA-seq, Genetics of Sex

## Abstract

Sex differences in gene expression have been widely studied in *Drosophila melanogaster*. Sex differences vary across strains, but many molecular studies focus on only a single strain, or on genes that show sexually dimorphic expression in many strains. How extensive variability is and whether this variability occurs among genes regulated by sex determination hierarchy terminal transcription factors is unknown. To address these questions, we examine differences in sexually dimorphic gene expression between two strains in Drosophila adult head tissues. We also examine gene expression in *doublesex* (*dsx*) mutant strains to determine which sex-differentially expressed genes are regulated by DSX, and the mode by which DSX regulates expression. We find substantial variation in sex-differential expression. The sets of genes with sexually dimorphic expression in each strain show little overlap. The prevalence of different DSX regulatory modes also varies between the two strains. Neither the patterns of DSX DNA occupancy, nor mode of DSX regulation explain why some genes show consistent sex-differential expression across strains. We find that the genes identified as regulated by DSX in this study are enriched with known sites of DSX DNA occupancy. Finally, we find that sex-differentially expressed genes and genes regulated by DSX are highly enriched on the fourth chromosome. These results provide insights into a more complete pool of potential DSX targets, as well as revealing the molecular flexibility of DSX regulation.

One major remaining question in biology is, ‘How does a shared genome give rise to two vastly different sexes?’ The Drosophila somatic sex determination hierarchy is responsible for directing sexual dimorphism in morphology, physiology, and adult behaviors (reviewed in Christiansen *et al.* 2002; Dauwalder 2011). Most sex-differential gene expression is expected to result from differences in how the terminal sex-specific transcription factors in this pathway regulate gene expression. How pervasive this regulation is, and how robust it is to genetic background and environmental differences, are open questions, especially given that both sex-differential expression and maleness/femaleness are quantitative traits.

In Drosophila, sex differences in gene expression have been widely studied in different tissues, and at different developmental time points (reviewed in Samson and Rabinow 2014). The sex determination hierarchy specifies sex differences in somatic tissues. The hierarchy consists of an alternative pre-mRNA splicing cascade, responsive to the number of X chromosomes, which directs the production of sex-specific transcription factors encoded by *doublesex* (*dsx*) and *fruitless* (*fru*) (Figure 1) (reviewed in Salz 2011). *dsx* establishes nearly all known morphological sex differences, and also has a role in the nervous system, whereas *fru* has a primary role in directing reproductive potential in the nervous system (reviewed in Manoli *et al.* 2006; Yamamoto and Koganezawa 2013).

**Figure 1:**
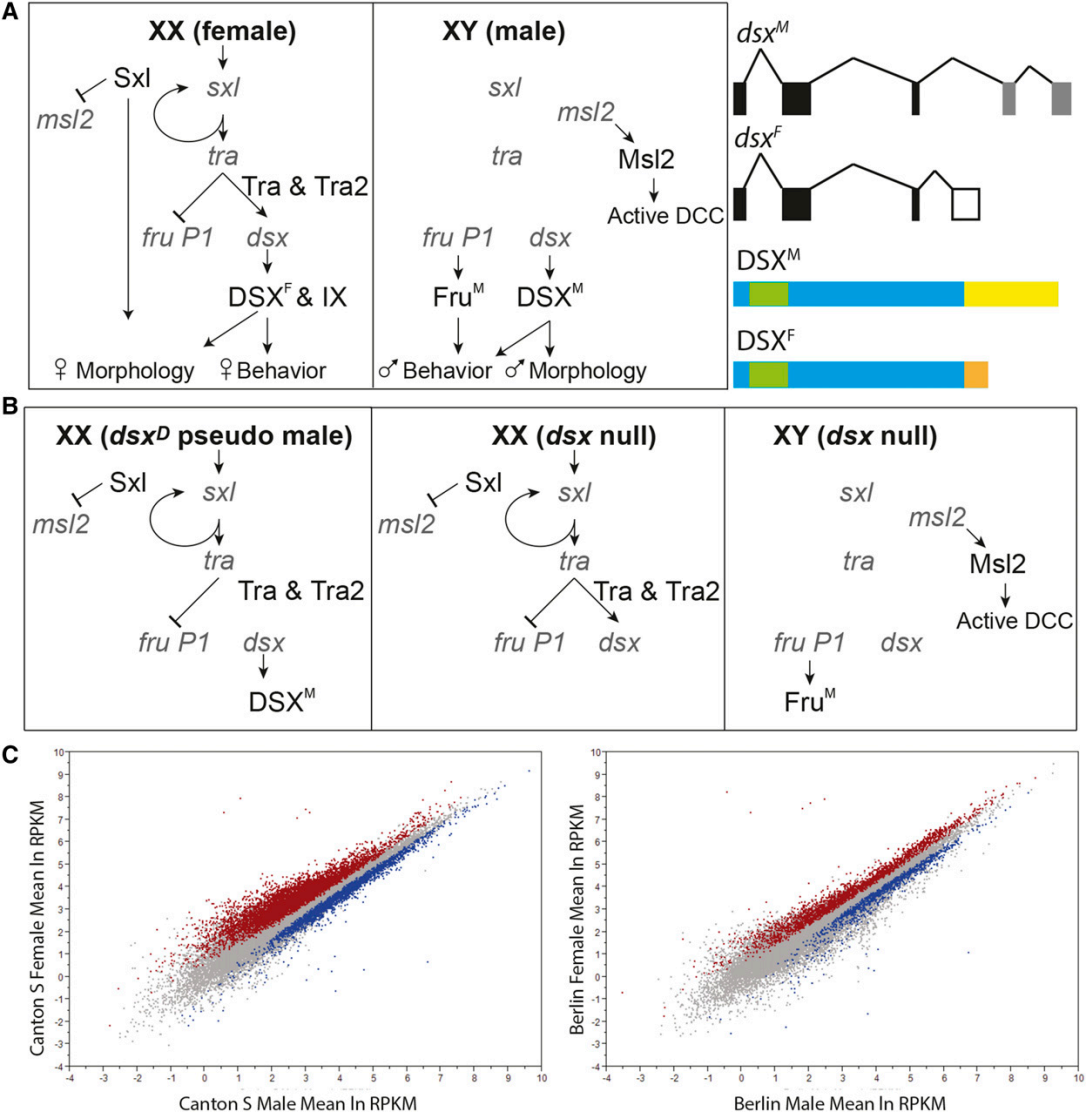
Drosophila sex determination hierarchy, effects of mutant alleles, and sex differences in expression in wild-type animals. (A) The *Drosophila* somatic sex determination hierarchy. The primary determinant of sex is the number of X chromosomes. The sex hierarchy includes sex-differentially produced splicing factors encoded by *sex-lethal* (*sxl*), *transformer* (*tra*), and splicing factor *transformer-2* (*tra-2*). Alternative splicing of *dsx* and *fru P1* pre-mRNAs leads to sex-specific production of DSX and Fru transcription factors. In females, dosage compensation (DCC) is not active due to the production of Sxl, which inhibits translation of *msl2*. In females, DSX^F^ together with IX regulate gene expression to direct female-specific behavior, morphology, and physiology. In males, DSX^M^ and Fru^M^ regulate gene expression to direct male-specific behavior, morphology, and physiology. Splicing differences of *dsx* pre-mRNAs, and schematics of the DSX protein isoforms are shown. The common DNA binding domain is indicated with a green box. The sex differences at the carboxyl-termini are shown in yellow and orange. (B) *dsx^D^* pseudo males are chromosomally XX, but produce only the male specific DSX isoform. Above *dsx*, the sex hierarchy is genetically the same as in wild-type females. *dsx* null animals do not have DSX produced, but above *dsx*, the hierarchy is genetically the same as the female and male wild-type animals. (C) Scatter plots of ln(RPKM) values for CS and Ber, with red (female) and blue (male) indicating statistically significant expression differences between the sexes.

In this study, we focus on the mechanisms used by *dsx* to generate sexual dimorphism in gene expression, and address the relationship between *dsx* regulation and consistent sex-differential gene expression across strains. We examine the global transcriptional profile of adult head somatic tissues in two wild-type strains, and in two *dsx* mutant strains. The *dsx*/*mab-3* (Dmrt) family of genes regulates sexual development across different taxa, and this role of the gene family appears to be an ancestral function (reviewed in Kopp 2012). Thus, this work could shed light on how quantitative differences between and among the sexes arise in diverse taxa.

In Drosophila, alternative sex-specific splicing of *dsx* pre-mRNAs generates male and female DSX isoforms (DSX^M^ and DSX^F^), which share a common amino-terminal DNA binding domain and bind the same DNA sequence motif, but differ in their carboxyl-terminal region (Figure 1) (Burtis and Baker 1989; Erdman and Burtis 1993). This difference allows DSX^F^ to interact with the product of *intersex* (*ix*), a homolog of a Mediator complex protein, which influences sex differences in DSX transcriptional activity (Garrett-Engele *et al.* 2002). Consistent with this, female *ix* mutants have the same intersexual phenotype as *dsx* null mutants, but male *ix* mutants do not (Baker and Ridge 1980).

Several genomic studies in Drosophila, as well as studies in other animals (reviewed in Ellegren and Parsch 2007), have suggested that sex differences in gene expression are pervasive. In fact, approximately 30–50% of the genome may show sexual dimorphism in transcript abundance (Jin *et al.* 2001; Arbeitman *et al.* 2002; Ranz *et al.* 2003; Gibson *et al.* 2004; Zhang *et al.* 2007; Graveley *et al.* 2011; Graze *et al.* 2014). However, in Drosophila, most of these differences are due to the presence of germline tissues, the ovary and testis (Arbeitman *et al.* 2002, 2004; Parisi *et al.* 2004; Goldman and Arbeitman 2007; Lebo *et al.* 2009; Catalan *et al.* 2012; Perry *et al.* 2014). Studies of sex differences in several somatic tissues have shown that far fewer genes show dimorphism in transcript abundance in somatic tissues, and that, for many of these genes, strain background has a large effect on sex-differential expression (Arbeitman *et al.* 2004; Tarone *et al.* 2005; Goldman and Arbeitman 2007; Lebo *et al.* 2009; Chatterjee *et al.* 2011; Catalan *et al.* 2012; Fear *et al.* 2015; Huylmans and Parsch 2015).

Given the importance of sex differences in the brain and other head tissues, the *a priori* expectation of many researchers has been that a sizeable proportion of genes expressed in the brain or head are regulated by *dsx* and *fru*, and that these genes should show large and consistent differences in expression between males and females. Several studies have identified sex-differentially expressed genes in these tissues, but these genes have varied across different studies (Goldman and Arbeitman 2007; Chang *et al.* 2011; Catalan *et al.* 2012). Disagreement across studies could result from differences in the strain used, tissues analyzed or experimental design. Assays of sex-differential expression in different strains, for the same tissue and using the same experimental design, have indicated that strain is an important factor. For example, in our previous genomic studies we identified genes with sex differences in expression that also showed sex-differential expression downstream of the sex hierarchy genes *transformer* and *dsx* (Figure 1 for hierarchy; and see Arbeitman *et al.* 2004; Goldman and Arbeitman 2007; Lebo *et al.* 2009; Chang *et al.* 2011). These latter studies required the sex differences to be present in different strain backgrounds. In all cases, this requirement substantially narrowed the starting list of genes identified with sex differences. Thus, these early studies of somatic gene expression differences identified genes with the most consistent sex hierarchy-dependent and strain-independent sex-differential expression. Additionally, previous direct examinations of variation across strains in sex-biased gene expression showed that strain background can have a significant effect (Tarone *et al.* 2005; Catalan *et al.* 2012; Huylmans and Parsch 2014).

Another contributing factor to differences observed across strains could be that DSX regulation appears to be flexible with respect to the regulatory mode by which sex-differential expression is produced. The regulatory mode could be associated with expression variation, if some modes produce more consistent sex-differential expression. Efforts to understand how DSX contributes to the generation of two different sexes have relied on early molecular-genetic studies of the *Yolk Protein* (*Yp*) genes, *Yp1* and *Yp2* (Baker and Ridge 1980; Bownes *et al.* 1983; Belote *et al.* 1985; Burtis *et al.* 1991). The *Yps* have highly female-biased expression in fat body tissue. It was shown that, in females, DSX^F^ activates *Yp* expression, whereas, in males, DSX^M^ represses *Yp* expression. These results suggest that sex differences are specified by sex-specific DSX isoforms having opposing roles. In one sex, sex-specific DSX activates expression of a set of genes, and, in the other sex, sex-specific DSX represses expression of these genes. Here, we call this mode of DSX regulation the opposing mode.

Subsequent genomic studies and gene-level validation studies showed that the opposing mode of DSX regulation is not the only mode (Arbeitman *et al.* 2004; Goldman and Arbeitman 2007; Lebo *et al.* 2009; Chatterjee *et al.* 2011; Luo and Baker 2015). Rather, additional modes were discovered that are more frequent (Arbeitman *et al.* 2004; Goldman and Arbeitman 2007; Lebo *et al.* 2009). The modes of DSX gene regulation, for a given gene, are: 1–4) DSX is an activator or repressor, but only in one sex; 5–6) DSX is either consistently an activator or a repressor in both sexes, but the extent of activation or repression is sex-specific; and 7) the opposing mode, for which *Yps* are the primary example. All modes were shown to be active in somatic tissues of the adult and in pupae (Arbeitman *et al.* 2004; Goldman and Arbeitman 2007; Lebo *et al.* 2009).

While the opposing mode of DSX gene regulation of *Yps* is highly consistent across strains, it is not clear if it is the DSX mode of regulation that leads to more consistent sex differences in expression across strain backgrounds. Furthermore, it is not clear if DSX DNA binding at a locus correlates with different DSX modes of gene regulation or influences consistent sex-differential gene expression across strains. The chromosomal locations of DSX targets are also underexplored; chromosomal bias has been observed for sex-differential expression and for regulatory targets of other sex regulatory hierarchy genes, such as *fru* (Parisi *et al.* 2003; Goldman and Arbeitman 2007; Chang *et al.* 2011; Catalan *et al.* 2012; Meisel *et al.* 2012; Dalton *et al.* 2013; Graze *et al.* 2014).

To address these questions we examined sex differences in gene expression in the head between males and females in two different wild-type laboratory strains: Canton-S (CS) and Berlin (Ber). To examine the modes of DSX regulation, we further examined sex differences in gene expression in chromosomally male and female *dsx* null mutants that have an intersexual phenotype, and in *dsx^D^/dsx^null^* chromosomally XX pseudo males—an allele combination in which only the DSX^M^ isoform is produced. We determined if the genes identified as regulated by DSX are enriched with those shown to be bound by DSX, using results from previous studies (Luo *et al.* 2011; Clough *et al.* 2014). Additionally, we determined if there is enrichment of DSX regulated genes on particular chromosome arms. Our results suggest that there is a large potential pool of DSX-regulated genes, with sex-differential expression dependent on strain, environment, and/or strain by environment interactions. Further, our results demonstrate that the fourth chromosome is enriched with DSX regulated genes.

## Materials and Methods

### Fly husbandry, tissue collection, and library preparation

Flies were raised on standard cornmeal food medium (33 l H_2_O, 237 g agar, 825 g dried deactivated yeast, 1560 g cornmeal, 3300 g dextrose, 52.5 g Tegosept in 270 ml 95% ethanol and 60 ml propionic acid). The incubator conditions are 25° on a 12-hr light:12-hr dark cycle.

Wild-type flies are the Canton-S and Berlin laboratory strains (obtained from U. Herberlein). Male (XY) and female (XX) *dsx* null flies are *dsx^d+r3^/dsx^m+r15^* (Duncan and Kaufman 1975; Baker *et al.* 1991). The *dsx^D^* pseudo males (XX) are *w,P{w^+mc^*, *Ubi-GFP}/+;dsx^D^*, *Sb^1^*, *e^1^/dsx^m+r15^* (for *dsx^D^* see Fung and Gowen 1957). The data for all experiments were generated at the same time, under the same environmental conditions. All flies were collected 0–16 hr post-eclosion under CO_2_ anesthetization, and allowed to recover for 8 hr before being snap-frozen in liquid nitrogen. All flies were stored at –80° until heads were collected. Adult heads were separated from the body by mechanical tapping in the cyrovial, and then separated while frozen on a piece of Plexiglass cooled on dry ice. Heads were immediately transferred to Trizol reagent. Libraries were prepared from three independent biological replicates for each condition. For each experimental condition, approximately 200 heads were used per library.

### Data processing and analysis

Illumina library preparation and read mapping to the *D. melanogaster* Release 5 genome, using FB5.30 annotation, was previously described (Dalton *et al.* 2013; Fear *et al.* 2015). To account for sex differences in transcript isoform expression level, expression was measured and analyzed at the exonic level (Supplemental Material, Figure S1). For each gene model, exons were classified as single or overlapping across isoforms (mapping following Graze *et al.* 2012). Overlap occurs among annotated exons of a gene when isoforms differ in start and end positions of exons corresponding to the same genomic region. Expression was measured for each single exon, and for each region of exonic overlap, considering the entire region of overlap. For simplicity, both cases are referred to as exons throughout.

Different gene models can also overlap, resulting in ambiguity with respect to gene expression. These regions are not considered in our analysis. Genes, and corresponding exons, that were not detected in all samples were also excluded from analysis. For an exon to be considered detected, it needed to have average per nucleotide coverage greater than zero in all samples. Overall, of 14,092 annotated genes, and 57,962 corresponding exons, 9673 genes and 42,602 exons showed detectable gene expression in head tissues for all samples. To allow comparisons, we considered only those genes with DSX DNA occupancy data (Luo *et al.* 2011; Clough *et al.* 2014). We analyzed 9476 genes (41,720 exons) in the final analysis.

Expression was normalized as the natural log of the number of reads per kilobase per million mapped reads (RPKM) per exon, and a linear model was fit (for RPKM, see Mortazavi *et al.* 2008). Using this model, contrasts were performed to detect differential expression between wild-type females and males, between wild-type females and *dsx^D^* pseudo males, and between each sex and the corresponding *dsx* null genotype. The adjusted FDR P-value was calculated considering all tests together and significance was considered at levels FDR < 0.05, < 0.10 and < 0.20 (Benjamini and Hochberg 1995). The FDR < 0.05 level was used in all cases, with the exception of Table 2. Analytical results are provided in Table S1.

Gene set enrichment analysis was conducted, using Fisher’s exact test, to determine if specific biological process, molecular function, or cellular component ontology terms are overrepresented among the set of genes corresponding to each DSX regulatory mode. For each of the biological process, molecular function, or cellular component ontologies, tests were performed with FDR correction for both strains and all modes considered together. Only genes with gene ontology (GO) annotation were considered in the enrichment analysis (Mootha *et al.* 2003; Rivals *et al.* 2007).

### Data availability

These data have been deposited in GEO. The accession number for wild-type strains is GSE50515. The accession number for the *dsx* mutant strains is GSE67400 (Dalton *et al.* 2013; Fear *et al.* 2015).

## Results

Here, we sought to understand the mechanism(s) that contribute to variability of sexually dimorphic gene expression across strains in the adult head (for example see Goldman and Arbeitman 2007). Sex differences in gene expression were analyzed at the exon level, to consider differences in transcript isoform abundance. When an exon for a gene is sex-differentially expressed, we also report differences at the gene level (Figure S1; and see Graze *et al.* 2012). Therefore, a gene with multiple transcript isoforms can be regulated by more than one DSX regulatory mode, depending on the exon/transcript isoform that is examined. Further, sex differences in particular transcript isoforms could be due to alternative pre-mRNA splicing, or differences in promoter deployment, which is not determined here (for example see Chang *et al.* 2011; Graveley *et al.* 2011). We examine sex-biased expression in two wild-type strains and *dsx* mutant strains, to identify the genes regulated by DSX, and to determine the mode of DSX regulation.

### Sex-differential expression of DSX-regulated genes varies across strains

We examined two strains from genetically distinct populations: the laboratory strains Canton-S (CS; from Canton, OH) and Berlin (Ber; from Berlin, Germany). Here, we find that CS and Ber have substantial differences in the total number of genes with sex-differential expression (Figure 1, Table 1, Table S2, and Table S3). We also find that the majority of sex differences in expression are strain-specific, with few genes showing consistently biased expression in the two strains, in agreement with previous reports (Arbeitman *et al.* 2004; Tarone *et al.* 2005; Goldman and Arbeitman 2007; Chang *et al.* 2011; Huylmans and Parsch 2015).

**Table 1:** Sex-differential expression in Canton S and Berlin strains

	Exons		Genes	
	WT Female to Male Comparison		WT Female to Male Comparison	
	Male-biased	Female-Biased	Male-Biased	Female-Biased
Canton S	2268	5686	1743	1672
Berlin	1070	2365	783	1611
Both	231	376	205	246
	WT Female to *dsx^D^* Comparison*^a^*		WT Female to *dsx^D^* Comparison*^a^*	
	Male-Biased	Female-Biased	Male-Biased	Female-Biased
Canton S	340	901	303	445
Berlin	78	207	69	168
Both	12	29	5	20
	WT to *dsx* Null Comparisons*^b^*		WT to *dsx* Null Comparisons*^b^*	
	Male-Biased	Female-Biased	Male-Biased	Female-Biased
Canton S	275	706	239	358
Berlin	52	120	44	105
Both	11	18	4	13

Each contrast compared the natural log of the RPKM normalized expression in wild-type females and males, wild-type females and *dsx^D^* pseudo males, and in wild-type females and *dsx* null females or wild-type males and *dsx* null males. A cut-off of FDR < 0.05 was used.

a For each exonic region, the wild-type comparison was considered first, and if sex-biased in the wild-type comparison, the exon was considered sex-differentially expressed and regulated by *dsx* if there was also significant sex-differential expression that was biased in the same direction in the wild-type female to *dsx^D^* comparison.

b If the exon was identified as a putative *dsx* target as in (*a*), then it was tested for a significant difference in either or both *dsx* null comparisons. Both exon level and corresponding gene level counts are reported.

To further examine the impact of *dsx* and strain background on sex-differential expression in each strain, we next identified exons that show sex-differential expression between males and females that are also regulated by *dsx* (Table 1), using data from *dsx^D^* pseudo males and *dsx* null genotypes. Here, the number of genes that meet the criteria for showing DSX regulation in all strains is substantially less than when we only considered sex-differential expression in each wild-type strain (Table 1), which could be due to strain background differences, or reflect lack of regulation by DSX.

### The seven modes of DSX regulation and differences between strains

**Figure 2:**
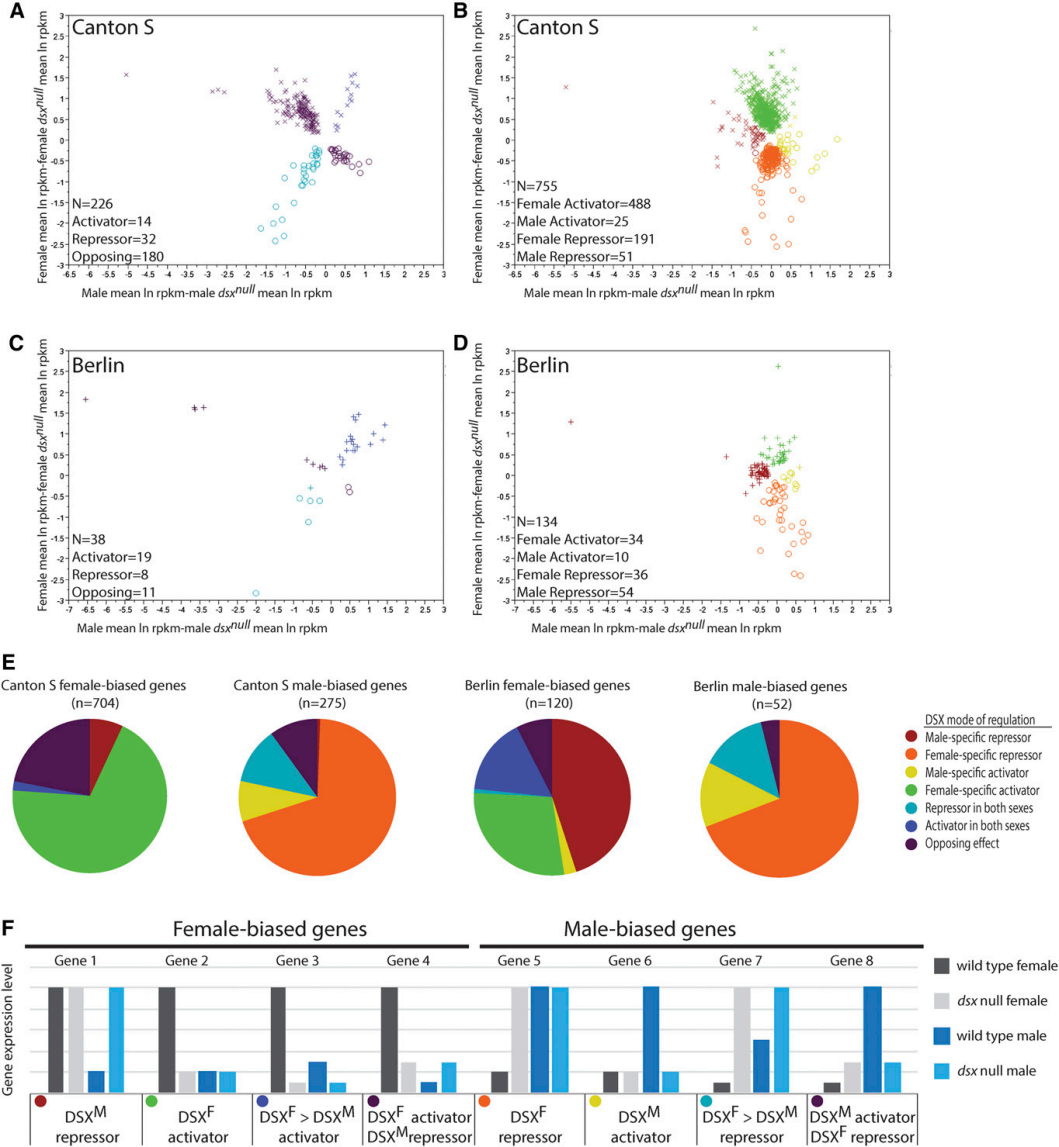
DSX modes of regulation and expression differences. The estimated expression differences between wild-type and *dsx* null animals are plotted for female comparisons by male comparisons [CS: (A) and (B); Ber: (C) and (D)]. In A–D male-biased genes (x) and female-biased genes (o) are indicated. (E) Pie charts showing the proportion of genes regulated by each DSX mode for female- and male-biased genes in CS and Ber. The legend on the right shows the colors used to indicate each DSX regulatory mode in this figure. (F) Hypothetical data demonstrating how DSX regulatory modes were determined (following Arbeitman *et al.* 2004; Goldman and Arbeitman 2007; Lebo *et al.* 2009). Expression in wild-type females or males of each strain was compared to expression in *dsx* null females or males, and both the significance of each test, and the direction of the mean difference in expression were considered. Thus, DSX can act as an activator or repressor in each sex, or both, and this defines the mode. If DSX^F^ activates expression in females, the expectation is that gene expression will be significantly lower in the absence of activation in *dsx* null females. Similarly, if DSX^F^ represses expression in females, gene expression is expected to be higher in the absence of DSX^F^ repression in *dsx* null females. Activation and repression were similarly examined in males. We note that the mode classification is sensitive to our ability to statistically detect expression differences.

Using the data from the *dsx* null comparisons, we are able to determine which DSX regulatory mode is responsible for sex-differential expression (Figure 2F). The plots in Figure 2, A–D show that, for most genes, the magnitude of expression differences between the wild-type and *dsx* null genotypes is similar (clustered symbols), with a few genes having very large effects of the *dsx* null genotype (symbols on the periphery; these include the *Yp* genes).

If we rank the DSX regulatory modes by number of genes that display regulation corresponding to each mode, we find that for male-biased genes the rank order between the two strains does not differ substantially. The most prevalent DSX regulatory mode for male-biased genes is female-specific repression (Figure 2E). An examination of the female-biased genes shows that the most prevalent regulatory mode in CS is female-specific activation by DSX, but in Ber it is male-specific repression by DSX. We previously found similar results on these DSX regulatory modes (Arbeitman *et al.* 2004; Goldman and Arbeitman 2007; Lebo *et al.* 2009). This suggests that there is regulatory flexibility in the generation of sex-differential gene expression, especially for genes with female-biased expression. While the opposing mode is the most well studied mode (darkest purple in Figure 2), it is not the most frequently observed mode for male- or female-biased genes.

**Figure 3:**
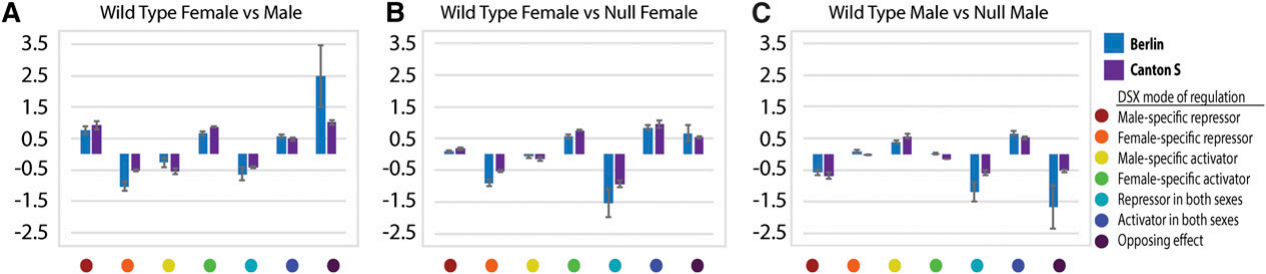
The effect of different DSX modes on regulation of expression. The estimate of differential expression for (A) wild-type females and males, (B) wild-type females and *dsx* null females, and (C) wild-type males and *dsx* null male comparisons, for each different DSX mode. The mean effect estimate, with standard error of the mean is shown for Ber (blue) and CS (purple) comparisons. The legend on the right shows the colors used to indicate each DSX regulatory mode.

We next determined if a particular regulatory mode gives rise to consistently larger estimates for sex-differential expression when we compare gene expression in either CS or Ber wild-type animals (Figure 3A). A positive estimate indicates female-biased expression, and a negative estimate male-biased expression. We also examined the size of expression differences in the *dsx* null comparisons (Figure 3, B and C), with a positive estimate in the *dsx* null comparisons indicating that DSX is an inducer and negative estimate indicating that DSX is a repressor. We do not observe large changes in the magnitude of expression differences, with none of the regulatory modes consistently generating larger sex differences in the two strains examined (Figure 3 and Table S4). This further suggests that there is flexibility in the relationship between DSX mode and the extent of sex-differential expression.

### DSX regulated genes and DSX DNA occupancy

We identified the set of genes that have the most consistent sex-differential expression, defined as those that are sex-differentially expressed in all strains used in this study and their DSX regulatory mode (Table 2, Table S5, and Table S6 for different FDR cut-off values). Of the most extensively validated DSX targets (*Yp* genes, *bab1* and *bab2*, and *Fmo-2*), both the *Yp*s and *Fmo-2* are identified as showing sex-differential expression that is consistent across strains (Table 2).

**Table 2:** DSX targets with consistent DSX regulation across strains

Gene	Chr.	Sex Bias	Regulatory Mode	DSX Occupancy*^a^*	DSX Occupancy*^b^*
CG8539	3L	Female	Activator	0	0
Cpr72Ea	3L	Female	Female specific activator	0	0
Mp20	2R	Female	Female specific activator	0	1
CG31522	3R	Female	Female specific activator	1	0
trpl	2R	Female	Male specific repressor	0	0
CG3759	2L	Female	Male specific repressor	1	0
**Yp2**	X	Female	Male specific repressor	1	1
**Fmo-2**	2R	Female	Male specific repressor	1	1
**Yp3**	X	Female	Opposing effects	0	1
**Yp1**	X	Female	Opposing effects	1	1
rost	2L	Female	Opposing effects/	1	1
			Male specific repressor		
CG15012	3L	Male	Female specific repressor	0	0
mav	4	Male	Female specific repressor	0	0
CG12158	2R	Male	Female specific repressor	0	0
Cyp4d21	2L	Male	Female specific repressor	0	0
CG31145	3R	Male	Female specific repressor	1	0
CG18547	3R	Male	Female specific repressor	1	0
Cyp313a1	3R	Male	Opposing effects	0	0

Genes with patterns of sex-differential expression in wild-type, *dsx^D^* pseudo male, and *dsx* null comparisons, and the same regulatory mode in both Canton S and Berlin strains (FDR < 0.10). Sex bias, the direction of sex-differential expression: Female (Female-biased) or Male (Male-biased); Regulatory mode, the seven types of DSX regulation; DSX occupancy.

a DSX^M^ and DSX^F^ ChIP-seq in S2 cells and DSX^M^ and DSX^F^ Dam-ID in female ovaries, and male and female fat body (Clough *et al.* 2014; Table S1).

b DSX^F^ Dam-ID in adult females (Luo *et al.* 2011; Table S1). Bold indicates validated DSX targets.

To understand the relationship between expression patterns and DSX DNA binding, we determined if the genes that have sex-differential expression are enriched for genes that are also bound by DSX, as identified in previous studies (Luo *et al.* 2011; Clough *et al.* 2014). We find that genes with validated occupancy of DSX, from both previous studies, significantly overlap with the gene lists identified as DSX-regulated genes in either CS or Ber (Table 3). Further, genes regulated by DSX are equally likely to be bound by DSX, irrespective of the mode of regulation (data not shown).

**Table 3:** DSX regulated genes and binding site occupancy

Strain	Test (Fisher’s Exact)	P-Value	Obs (Exp)	Fold Enrichment
Canton S	Luo *et al.* 2011 Occupancy	<0.0001	62 (30)	2.07
Canton S	Clough *et al.* 2014 Occupancy	<0.0001	355 (198)	1.79
Berlin	Luo *et al.* 2011 Occupancy	<0.0001	21 (8)	2.63
Berlin	Clough *et al.* 2014 Occupancy	0.0008	69 (49)	1.41

Fisher’s exact test was performed to detect enrichment or depletion of genes with previously observed DSX^M^/DSX^F^ occupancy in ovaries, fat body and in S2 cells (Clough *et al.* 2014), or in adult female flies (DSX^F^; Luo *et al.* 2011) among genes identified as regulated by *dsx* in 8- to 24-hr-old male or female heads (this study). While this study examines *dsx* regulation in males and females, the fold enrichment of genes with DSX occupancy in adult female flies (Luo *et al.* 2011) is greater than the fold enrichment of genes identified in the Clough *et al.* (2014) study.

We also determined if sex-differentially expressed genes are more likely to reside on different chromosome arms. Previous studies have shown that genes with male-biased expression in adult head tissues are enriched on the X chromosome (Goldman and Arbeitman 2007; Chang *et al.* 2011; Catalan *et al.* 2012), whereas other studies did not find a similar enrichment (discussed in Huylmans and Parsch 2015). Further analyses reconciled this conflicting observation, noting that the enrichment on the X of genes with male-biased expression in the head was likely due to expression in the central nervous system, and might be at the limit of detection in whole head studies (Huylmans and Parsch 2015). Here, we find a significant enrichment of sex-biased genes identified in CS on the X chromosome. We also find that the fourth chromosome is both significantly enriched for genes with sex-differential expression (Table 4 and Table S7) and significantly enriched for genes with validated occupancy by DSX (Table 5).

**Table 4:** Sex-differential expression and chromosome bias

Chr.	Strain	Sex-Differentially Expressed			DSX-Regulated		
		P-Value	Obs (Exp)	Fold	P-Value	Obs (Exp)	Fold
X	Canton S	0.02	587 (547)	1.07	N.S.	144 (126)	1.14
2L	Canton S	N.S.	587 (569)	1.03	N.S.	129 (131)	0.98
2R	Canton S	0.03	625 (665)	0.94	N.S.	138 (153)	0.90
3L	Canton S	N.S.	599 (632)	0.95	N.S.	143 (146)	0.98
3R	Canton S	N.S.	774 (790)	0.97	N.S.	170 (182)	0.93
4	Canton S	< 0.0001	63 (27)	2.33	< 0.0001	21 (6)	3.50
X	Berlin	0.01	436 (397)	1.10	N.S.	39 (40)	0.98
2L	Berlin	N.S.	423 (413)	1.02	N.S.	46 (42)	1.09
2R	Berlin	N.S.	511 (483)	1.06	0.01	65 (49)	1.33
3L	Berlin	0.0003	398 (458)	0.87	N.S.	38 (46)	0.83
3R	Berlin	0.02	531 (573)	0.93	N.S.	46 (58)	0.79
4	Berlin	< 0.0001	46 (19)	2.42	*N.S.*	2 (2)	1.00

Fisher’s exact test was performed to detect enrichment or depletion of genes identified as sex-differentially expressed or genes identified as regulated by *dsx*, here, on each chromosome arm. The observed (Obs) and expected (Exp) number of genes are reported for each major chromosome arm, as well as the fold enrichment (Fold).

**Table 5:** Chromosome bias in DSX occupancy

Occupancy	Chr.	Obs (Exp)	Fold	P-Value
Clough *et al.* 2014	X	553 (530)	1.04	N.S.
	2L	579 (551)	1.05	N.S.
	2R	619 (644)	0.96	N.S.
	3L	617 (612)	1.01	N.S.
	3R	727 (766)	0.95	N.S.
	4	43 (26)	1.65	< 0.0001
Luo *et al.* 2011	X	76 (81)	0.94	N.S.
	2L	84 (84)	1.00	N.S.
	2R	95 (98)	0.97	N.S.
	3L	113 (93)	1.22	0.024
	3R	107 (117)	0.91	N.S.
	4	4 (4)	1.00	N.S.

Fisher’s exact test was performed to detect enrichment or depletion of genes showing evidence of DSX occupancy in Clough *et al.* 2014, or in Luo *et al.* 2011, on each chromosome arm. The observed (Obs) and expected (Exp) number of genes are reported for each major chromosome arm, as well as the fold enrichment (Fold).

## Discussion

The results of this study further confirm that sex differences in gene expression can show substantial differences across strains. While we found different sets of genes with sex-differential expression in each strain, the gene sets from both strains were highly significantly enriched for DSX DNA occupancy, suggesting that they are *bona fide* targets. There are many genes that have been implicated in being regulated by DSX using independent methodologies, including gene expression studies and direct observation of DSX DNA occupancy at specific loci. If one considers all of these studies and the observed differences across the studies, it suggests that there is a pool of DSX-regulated genes with the potential for sex-differential expression, but whether they will show sex-differential expression depends on strain, environment, and strain by environment interactions. This is the case even when the same tissue and developmental time points are examined. Perhaps having this pool of potential targets is one mechanism contributing to natural variation in gene expression, resulting in quantitative differences in maleness/femaleness among individuals in a population.

Given the seven different modes of DSX regulation, we wanted to determine if a particular DSX regulatory mode is responsible for the strain differences we observed. We hypothesized that some modes would show less consistency in generating sex-differential expression across the strains. The male-biased genes identified in CS and Ber had a similar rank order with respect to the number of genes regulated by a DSX mode, whereas female-biased genes did not. The results suggest that the regulatory modes to generate female-biased expression might be more flexible at a molecular level, but we found that all DSX modes of regulation are similarly sensitive to strain background. Further, we did not see clearly defined functional differences in the genes downstream of each DSX regulatory mode, on the basis of gene set enrichment analyses of ontologies (Table S8).

The difference in sex-differential expression between the two wild-type strains considered was striking. Why does CS display more significant sexual dimorphism of gene expression, as compared to Ber? It is possible that this is a consequence of their original genetic background or it could be due to differences in the conditions in which they have been reared, resulting in different selective pressures. It appears that both of these strains were initially collected in the wild and reared as laboratory strains beginning in the 1930s (Argelander and Strasburger 1939; Lindsley 1983; deBelle and Heisenberg 1996), though there is no clear documented lineage for the Ber wild-type strains in current use. It will be interesting to determine if variation in sex-differential expression is related to geographical region, the length of time strains have been reared in the laboratory, or differences in rearing conditions over time.

What are the molecular mechanisms driving strain differences? It is clear that sex-differential expression in the head is both dynamic across strains and genetically specified, with the molecular mechanisms that generate this type of gene regulation under active investigation. For example, studies of DSX *cis*-regulation of *bab*, *Fad2* and *fmo-1* show polymorphism in *cis*-regulatory elements can confer novel patterns of *dsx*-directed sexually dimorphic expression, while retaining ancestral monomorphic expression patterns that are essential in development (Kopp *et al.* 2000; Williams *et al.* 2008; Shirangi *et al.* 2009; Rogers *et al.* 2013; Luo and Baker 2015). It is unclear how similar *cis* polymorphism in DSX binding sites and associated DNA regions mediate natural variation in expression at the population level, but *cis* variation may contribute to the strain differences observed.

It is also possible that strain differences in sex-differential expression can be accounted for by strain differences in *trans* factors. This includes the abundance and/or splicing of sex hierarchy and cofactor mRNAs, such as those encoded by *sxl*, *tra*, *tra-2*, *ix*, *hermaphrodite* and/or dosage compensation genes, as was previously examined (Tarone *et al.* 2005; Fear *et al.* 2015; Li *et al.* 2015). An examination of expression levels for these genes in CS and Ber data did not reveal apparent differences in expression level that could account for the differences observed. Differences in biochemical activity, independent of expression levels, could also contribute to variation, including nonlinear responses due to differences in transcription factor binding affinity, or number of binding events, splicing efficiency, and co-factor protein interactions, as previously proposed (Tarone *et al.* 2005, 2012). In the case of *dsx*, differences in splicing efficiency could result in some cells expressing both the male- and female-specific DSX isoforms, which would influence DSX biochemical and regulatory activity, as DSX functions as a dimer (Cho and Wensink 1997). Previous loss-of-function and gain-of-function studies on DSX have suggested that the differences between the sexes for many phenotypes are a result of DSX functioning more like a dial than a switch (Jursnich and Burtis 1993). Our results are consistent with that idea, and go further to show that quantitative differences in regulation of DSX targets across strains are a likely mechanism contributing to natural variation in sexual dimorphism.

We note that DSX DNA binding does not appear to be sufficient to explain all DSX-regulated differences, as one of the studies examining DSX DNA occupancy, showed that DSX can be bound to a locus, independent of whether the gene is sex-differentially expressed (Clough *et al.* 2014). This suggests that more complex *cis*, *trans*, and *cis* by *trans* regulatory effects need to be considered for a more complete understanding of variation across strains. Future analyses will also benefit from a more thorough understanding of the nucleotide variation across strains, especially in DSX binding sites and their surrounding DNA, DSX transcriptional co-factors and deeper knowledge of the mechanisms of gene regulatory logic.

Our results further the idea that large-scale differences in interactions across branches of the sex hierarchy contribute to sex-differential expression. In previous work, we have suggested that sex-specific transcription factors may take advantage of the unique male and female nuclear environments, with dosage compensation or sex chromosome differences contributing to differences between males and females through interactions with sex hierarchy transcription factors (Goldman and Arbeitman 2007; Chang *et al.* 2011; Dalton *et al.* 2013). This idea can be extended in light of our observation that the fourth chromosome has a significant enrichment of genes identified as sex-differentially expressed in this study, and the fourth chromosome is also significantly enriched with genes with known DSX occupancy.

Interestingly, there is evidence that the Drosophila autosomal fourth chromosome evolved from an ancestral sex chromosome (Vicoso and Bachtrog 2013), and that regulation on the fourth and X chromosome share components of the dosage compensation machinery (Lundberg *et al.* 2013). Indeed, studies examining embryos and gonadal tissues, ovary and testis, showed an excess of female-biased expression and genes expressed in the ovary on the fourth chromosome in both *D. melanogaster* and in distantly related Brachyceran species, for which chromosome 4 (Muller’s F element) is a sex chromosome (Vicoso and Bachtrog 2013).

Regulation of sexually dimorphic expression by DSX and Fru, and their associated sex hierarchy splicing factors, predate the evolutionary turnover of sex chromosomes in Diptera (Davis *et al.* 2000; Kopp 2012). Furthermore, enrichment of sex-differential expression on sex chromosomes is thought to occur due to the unique molecular and evolutionary properties of sex chromosomes (reviewed in Vicoso and Charlesworth 2006; Ellegren and Parsch 2007). Thus, identifying targets of DSX and Fru and their chromosomal locations in a wider range of insect species may yield new insights into the molecular basis and evolution of sex-biased gene expression on sex chromosomes.

Sex differences in expression in the head were small in magnitude in many cases. Examining other tissues with larger effects will be important in the future. We also note that the *dsx* mutant alleles analyzed here are in different strain backgrounds than CS and Ber, and this could also influence our detection of the different DSX regulatory modes in CS and Ber. Furthermore, differences in fecundity are an important factor to consider, especially since the *dsx* mutants are sterile, and there are known tissue-interactions that influence gene expression in the adult head (Parisi *et al.* 2010). To fully understand variation in expression, future gene-level molecular analyses of tissue specific, cell-type specific or single-cell regulation, as well as developmental studies, will be important.

Wild animals experience different environmental conditions and are generally outbred. Thus, it is unclear *a priori* what will be a male- or female-biased gene in a wild population, because the sex-biased expression could vary both qualitatively and quantitatively. This type of regulatory variation may occur in other species and contribute to natural variation in sexual dimorphism. Indeed, our studies showed there is substantial variation in sexually dimorphic gene expression in the adult mouse hippocampus across strains (Vied *et al.* 2016). Our results also suggest that strain differences across studies may be contributing to the lack of reproducibility of some genomic studies, an important consideration as this issue is being widely evaluated in the scientific community (Editorial 2013). In the future, it will be interesting to perform similar studies at the population level, with an experimental design that allows one to ascertain *cis*, *trans*, and *cis* by *trans* regulatory effects, as well as population structure effects. In addition, understanding the molecular basis for variability of gene expression and flexibility in transcription factor regulatory mode, as we see for *dsx* regulated genes, is important in understanding how quantitative differences in femaleness/maleness arise.

## Supplementary Material

Supplemental Material
