# Supplementary material for: Sex Differences in Drosophila Somatic Gene Expression: Variation and Regulation by doublesex
Source: G3 (Bethesda). 2016 Apr 19;6(7):1799–808. doi: 10.1534/g3.116.027961 (PMC4938635; doi:10.1534/g3.116.027961)
Supplement: Supplemental Material [file supp_6_7_1799__index.html]

Sex Differences in Drosophila Somatic Gene Expression: Variation and Regulation by doublesex — Supplemental Material 

# Sex Differences in *Drosophila* Somatic Gene Expression: Variation and Regulation by *doublesex*

## Supplemental Material for Arbeitman *et al.*, 2016

**Files in this Data Supplement:**

- Figure S1 - Illustration of exonic regions and nomenclature. (.ai, 1103 KB)
- Table S1 - Results for all analyzed exons (sheet A). (.xlsx, 6 MB)
- Table S2 - Exons (A) with patterns of sex-differential expression in wild type comparisons (FDR < 0.05). (.xlsx, 3705 KB)
- Table S3 - Exons (A) with patterns of sex-differential expression in wild type and in female to *dsxD* pseudomale comparisons (FDR < 0.05). (.xlsx, 1469 KB)
- Table S4 - Summary statistics for the estimated difference in expression for all genes with sex-differences in expression in wildtype, *dsxD* and *dsx* null comparisons (FDR < .05). (.xlsx, 105 KB)
- Table S5 - Genes with patterns of sex-differential expression in wild type, *dsxD* pseudomale and *dsx* null comparisons and with the same regulatory mode in both Canton S and Berlin strains (FDR < 0.05). (.xlsx, 48 KB)
- Table S6 - Genes with patterns of sex-differential expression in wild type, *dsxD* pseudomale and *dsx* null comparisons and with the same regulatory mode in both Canton S and Berlin strains (FDR < 0.20). (.xlsx, 66 KB)
- Table S7 - Genes with sex-differential expression (male- and female-biased) and chromosome bias. (.xlsx, 12 KB)
- Table S8 - GO term enrichments (p < 0.05) for Biological Process (A), Molecular Function (B) and Cellular Process terms (C). (.xlsx, 15 KB)
