# Supplementary material for: Sex Differences in Drosophila Somatic Gene Expression: Variation and Regulation by doublesex
Source: G3 (Bethesda). 2016 Apr 19;6(7):1799–808. doi: 10.1534/g3.116.027961 (PMC4938635; doi:10.1534/g3.116.027961)
Supplement: Supplemental Material [file supp_g3.116.027961_FigureS1.ps]

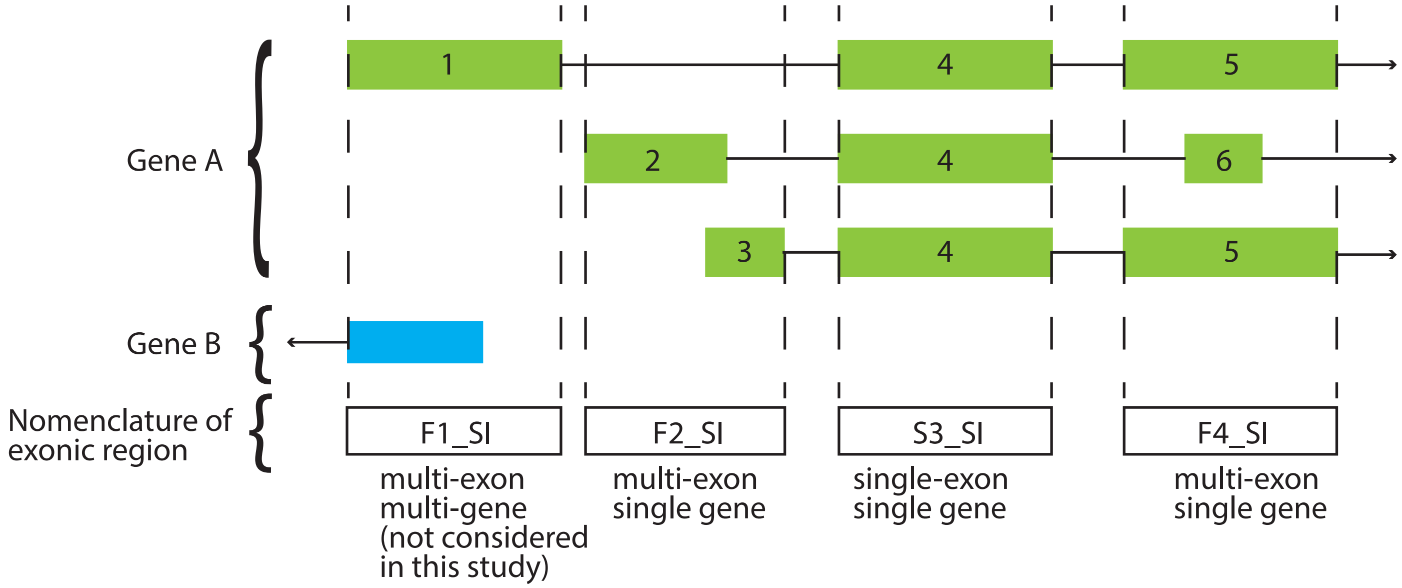

Supplemental Figure 1: Illustration of exonic regions and nomenclature.

Exonic regions were classified as belonging to either a single exon (S#_SI), or mutiple overlapping exons, F#_SI.

An F designation indicates that there are overlapping exons from either the same gene or different genes. In this study only fusions from single genes are considered, due to the ambiguity of assigning reads.

An S desingation indicates that there are no overlapping exons.
